# Properties of the epileptiform activity in the cingulate cortex of a mouse model of *LIS1* dysfunction

**DOI:** 10.1007/s00429-022-02458-1

**Published:** 2022-02-01

**Authors:** E. Domínguez-Sala, A. Andreu-Cervera, P. Martín-Climent, R. Murcia-Ramón, S. Martínez, Emilio Geijo-Barrientos

**Affiliations:** 1 Instituto de Neurociencias, Universidad Miguel Hernández-CSIC, Campus de San Juan, C/Ramón y Cajal sn, San Juan de Alicante, 03550 Alicante, Spain; 2 Centro de Investigación Biomédica en Red en Salud Mental (CIBERSAM, Spain), Madrid, Spain

**Keywords:** Lissencephaly, AMPA receptors, Epileptiform activity, Anterior cingulate cortex, Retrosplenial cortex, Cortical circuits

## Abstract

Dysfunction of the *LIS1* gene causes lissencephaly, a drastic neurological disorder characterized by a deep disruption of the cortical structure. We aim to uncover alterations of the cortical neuronal networks related with the propagation of epileptiform activity in the *Lis1/sLis1* mouse, a model lacking the LisH domain in heterozygosis. We did extracellular field-potential and intracellular recordings in brain slices of the anterior cingulate cortex (ACC) or the retrosplenial cortex (RSC) to study epileptiform activity evoked in the presence of bicuculline (10 µM), a blocker of GABA_A_ receptors. The sensitivity to bicuculline of the generation of epileptiform discharges was similar in wild type (WT) and *Lis1/sLis1* cortex (EC_50_ 1.99 and 2.24 µM, respectively). In the *Lis1/sLis1* cortex, we observed a decreased frequency of the oscillatory post-discharges of the epileptiform events; also, the propagation of epileptiform events along layer 2/3 was slower in the *Lis1/sLis1* cortex (WT 47.69 ± 2.16 mm/s, *n* = 25; *Lis1/sLis1* 37.34 ± 2.43 mm/s, *n* = 15; *p* = 0.004). The intrinsic electrophysiological properties of layer 2/3 pyramidal neurons were similar in WT and *Lis1/sLis1* cortex, but the frequency of the spontaneous EPSCs was lower and their peak amplitude higher in *Lis1/sLis1* pyramidal neurons. Finally, the propagation of epileptiform activity was differently affected by AMPA receptor blockers: CNQX had a larger effect in both ACC and RSC while GYKI53655 had a larger effect only in the ACC in the WT and *Lis1/sLis1* cortex. All these changes indicate that the dysfunction of the *LIS1* gene causes abnormalities in the properties of epileptiform discharges and in their propagation along the layer 2/3 in the anterior cingulate cortex and in the restrosplenial cortex.

## Introduction

LIS1 is a 46 kDa protein involved in synapse formation, synapse modulation, and neuronal migration, which plays a crucial role not only during brain development but also in adult stage. LIS1 is encoded by *PAFAH1B1* gene, located in the chromosome 17, and formed by two functional domains. LisH domain is located in the N-terminus portion and mediates the interaction with other LIS1 peptides to produce a LIS1 homodimer, the functional form of the protein. The following portions are a coiled-coil domain with structural function and WD40 domain, sited in the C-terminus, which mediates the interaction with target proteins (Reiner et al. 2002). LIS1 is a regulator of the binding of dynein to the microtubules. Dynein is a molecular motor which regulates the dynamics and stability of microtubules during cell growth and movement, and also with the transport of cargos along them. These mechanisms are the ones supposed to be altered in classic individuals with lissencephaly since they are directly involved in neuronal migration, axon growth and synapse formation and also other partners from the same cascade, such as DXC, produce other forms of lissencephaly with similar pathophysiology (Wynshaw-Boris and Gambello 2001). However, LIS1 also acts as a non-catalytic regulatory subunit of the acetyl-hydrolase of the platelet activating factor (PAF-AH), which regulates the levels of the platelet aggregation factor (PAF) (Hattori et al. 1994). Some studies also suggest that PAF can be involved in neuronal migration and synaptic modulation (Clark et al. 1992; Gopal et al. 2009).

Alterations in the encoding and regulatory sequence of *LIS1* have been largely described as the cause of classic lissencephaly, a drastic neurological disorder characterized by the alteration of number and size of brain gyri and sulci (pachygyria) or its complete absence (agyria) (Lo Nigro et al. 1997). One hallmark of the *LIS1* dysfunction is the deep disorganization of the cortical structure, which is commonly reflected in the loss of the typical six-layering pattern due to disrupted neuronal migration during developmental stages. On the other hand, the functional hallmark of classic lissencephalic brains is the recurrent apparition of intractable and fast-propagated epileptic seizures, which produce a progressive cognitive decline, compromising the quality of life and life expectancy of the patients (Dobyns 1993).

A wide variety of alterations in *LIS1* have been described in different cohorts of classic lissencephaly patients (Saillour et al. 2009), ranging from point mutations to prominent deletions affecting encoding or regulatory regions of the gene; however, the relationship among different alterations in the LIS1 protein and the pathophysiology of the classic lissencephaly is not fully understood. Previous investigations in murine models have revealed some details about how neuronal networks are affected by LIS1 dysfunction. Extracellular recordings “in vivo” in a murine model of *LIS1* dysfunction showed that a 50% reduction in the LIS1 protein dosage produced an increase of excitability in several hippocampal networks, such as the dentate gyrus and the CA1 area, leading to the appearance of epileptic-like activity in both regions (Fleck et al. 2000; Dinday et al. 2018). Additional work in the same model revealed that this over-excitation is the consequence of an increased probability of release of glutamate vesicles from perforant pathway presynaptic terminals on granule cells of the dentate gyrus, and from Schaffer collaterals on CA1 pyramidal neurons (Greenwood et al. 2009; Hunt et al. 2012). This loss of excitatory control might be produced by the overall disruption of the cellular structure of these hippocampal regions, given that many glutamatergic and GABAergic neurons were ectopically positioned as a result of aberrant neuronal migration during the development (Hirotsune et al. 1998; Fleck et al. 2000; Wang and Baraban 2007; Jones and Baraban 2007).

In the present work, we study the properties of synchronic epileptiform activity evoked by inhibition of GABA_A_ receptors in the neocortex of the *Lis1/sLis1* mice. This mouse is a model of LIS1 dysfunction which presents a complete deletion of the LisH domain (Cahana et al. 2001); this LIS1 mutation is similar to that of a lissencephaly patient with an in-frame N-terminal deletion (Fogli et al. 1999), but it is different from the mutations of the Lis1 ± mouse described by Hirotsune et al. (1998); the developmental alterations detected in the *Lis1/sLis1* and Lis1 ± models are also different (Cahana et al. 2001; Hirotsune et al. 1998). In the *Lis1/sLis1* model, it has been shown the presence of abnormalities in the inhibitory synaptic transmission in the cerebral cortex, including alterations in GABA_A_ receptors (Valdés-Sánchez et al. 2007). Our objective is to study the properties and propagation of epileptiform discharges along layer 2/3 in the disinhibited cingulate cortex of the *Lis1/sLis1* model. The experiments were done using electrophysiological recordings in brain slices of *Lis1/sLis1* and wild type animals. Our results indicate that the lack of LisH in one allele produces a clear slowing down of the propagation of the epileptiform activity along the layer 2/3 of the anterior cingulate and the retrosplenial cortex, and suggest that abnormalities of the synaptic transmission mediated by glutamate receptors could be implicated in this effect. We show also that there are no differences between wild type and mutant animals in the sensitivity to bicuculline of the generation of epileptiform discharges, and that there are abnormalities of the spontaneous EPSCs recorded in pyramidal neurons of mutant animals (lower frequency and higher peak amplitude). Overall, these results indicate that the dysfunction of the *LIS1* gene causes abnormalities in the properties of epileptiform discharges and in their propagation along the layer 2/3 of these cortical areas.

## Methods

### Animals and slice preparation

Experiments were done in brain slices prepared from male *Lis1/sLis1* mice (Cahana et al. 2001). Mice (14–16 postnatal days) were maintained, managed, and sacrificed following the international laws and policies (Spanish Directive “RealDecreto1201/2005”; European Union Directive 2010/63/UE). All protocols were approved by the Ethical Committee for Experimental Research of the Universidad Miguel Hernández (code: 2018/VSC/PEA/0035). Mice were genotyped before performing the experiments by PCR. A ≈ 750 bp DNA fragment was amplified from mutant mice tissue sample lysate with the following primers: 3’GGTGGCAGTGTTGAGATG CCTAGCC5’ and 5´GCATTCCTGTAATCCAGTACCTGG 3´. Amplification was performed for 35 cycles with single initial denaturalization step of 94° for 5 min. Each cycle was composed by a denaturalization step of 94° for 40 s, hybridization step of 60° for 45 s and a polymerization step of 72° for 10 s.

Animals were killed by cervical dislocation, decapitated, and their brains were quickly removed and sliced (400 µm, coronal plane) with a vibratome (LeicaVT1000) submerged in ice-cold cutting solution (composition in mM: NaCl, 124; KCl, 2.5; PO4H2Na, 1.25; MgCl_2_, 2.5; CaCl_2_, 0.5; NaCO_3_H, 26; glucose, 10; pH 7.4 when saturated with 95% O_2_ and 5% CO_2_). The slices were transferred to a modified artificial cerebro-spinal fluid (mACSF) with high extracellular potassium for enhanced excitability (composition in mM: NaCl, 124; KCl, 5; PO4H2Na, 1.25; MgCl_2_,1; CaCl_2_, 1.2; NaCO_3_H, 26; glucose, 10; pH 7.4 when saturated with 95% O_2_ and 5% CO_2_); slices were incubated in this solution at 37 ºC during 30 min. and then remained at room temperature until recordings; recordings were obtained at least 60 min after preparing the slices. Anterior cingulate cortex (ACC) and retrosplenial cortex (RSC) slices were selected by their position along the rostro-caudal axis according to the mouse brain atlas by Paxinos and Franklin (2001). We considered ACC slices those placed between 1.10 and 0.14 mm from the bregma, and RSC slices those placed between − 1.06 and − 2.06 mm from the bregma.

### Electrophysiological recordings

For recording, one slice was placed in the recording chamber where it was perfused with mACSF at ≈3 ml/min (33–34 °C). For extracellular recordings, bicuculline (10 µM) was added to the ACSF (except in the experiments of bicuculline dose–response relationship shown in Fig. 2). Extracellular recordings were done with glass pipettes pulled of borosilicate glass (1.5 mm o.d., 0.86 mm i.d., with inner filament) and filled with mACSF (3–6 MOhm). In each slice a single electrode was successively placed in different recording positions within layer 2/3 to obtain electrophysiological recordings, as shown in the Results section. Local field potentials were recorded with a MultiClamp 700B amplifier (Axon Instruments, Molecular Devices, USA), filtered at 5 kHz and digitized at 20 kHz (Digidata 1200B or 1440A Axon Instruments, Molecular Devices, USA). Bicuculline, GYKI53655, cyclothiazide, and CNQX were obtained from Sigma–Aldrich (USA). Extracellularly recorded responses in layer 2/3 were evoked by applying electrical stimuli to layer 1 using bipolar concentric electrodes. Responses were evoked by square current pulses (0.1 ms of duration) whose amplitude was adjusted to 2 × the threshold intensity. The latency of the evoked responses was calculated from the onset of the stimulus artifact to the time at which the response was 10% of its peak amplitude. The frequency of the oscillatory post-discharges was measured from the autocorrelogram computed during an interval of 250–500 ms of regular oscillatory activity; the frequency was measured from the interval between the 1st (at 0 ms, with the highest autocorrelation) and the 2nd peaks of the autocorrelogram (see below, Fig. 3).

Single neuron electrophysiological parameters were studied using somatic whole-cell intracellular recordings from pyramidal neurons in layer 2/3 of the dorsal agranular RSC (aRSC). Pyramidal neurons were selected by the shape of the soma and the prominent apical dendrite as seen under the microscope with DIC optics. Intracellular recordings were obtained with patch pipettes made of borosilicate glass that had 3–5 MOhms when filled with the intracellular solution (composition in mM: 130 potassium gluconate, 5 KCl, 5 NaCl, 5 EGTA, 10 HEPES, 2 Mg-ATP, 0.2 Na-GTP; pH 7.2 adjusted with KOH; 285–295 mOsm). Resting membrane potential was measured after achieve whole-cell configuration. Series resistance was compensated in current-clamp mode with the bridge balance tool of the Multiclamp. Passive responses and action potential firing were obtained in current-clamp configuration by applying a protocol of consecutive current square pulses (− 150 pA to 200 pA; 1 s of duration, 25 pA of increment). Membrane input resistance was calculated from the voltage deflection caused by small (− 25 to − 50 pA) hyperpolarizing current pulses. Action potential (AP) threshold was estimated as the potential level at which there was a clear abrupt increase in the slope. AP amplitude was measured from action potential threshold. AP duration was measured at 50% from the AP peak. Action potential after-hyperpolarization (AHP) peak amplitude was measured from AP threshold. AHP duration was measured from the peak to the 50% of the peak amplitude. Spontaneous postsynaptic excitatory currents (sEPSCs) were recorded in voltage-clamp configuration, holding the membrane potential of the cell at − 68 mV (equilibrium potential for Cl–- calculated from the extra and intracellular concentrations). Spontaneous synaptic currents detection and analysis were performed using WinEDR 3.9.1 software (Dr. J. Dempster, University of Strathclyde, Glasgow UK). All drugs used in the experiments were added to the mACSF at the specified concentrations from concentrated stocks prepared in water (bicuculline, GYKI53655, and CNQX; stocks at 1000 × the final maximum concentration) or in DMSO (cyclothiazide; stock 50 mM).

### Lentivirus injection and immunofluorescence

Postnatal P4 mice were anesthetized and injected with a lentivirus that expresses GFP under the promotor of CaM kinase IIa (Lv-CkIIa-GFP; SignaGene; SL100307). At P14, the injected and non-injected mice were perfused with paraformaldehyde (PFA) 4% in PBS, and the brains were maintained in PFA 4% overnight at 4 ºC. The brains were embedded in agarose at 4% and cut in slices using a vibratome (100 µm of thickness). The slices were incubated in PBST with 1% of BSA and 10% of lysine and incubated with the primary antibodies overnight at 4ºC: chicken anti GFP (1:200, AVES; GFP-1020), mouse anti GluA2 (1:500, Fisher; 32-030-0), mouse anti-GluA1 (1:500, Rockland, 200-301-D61), rabbit anti-GluK5 (1:500, Abcam, ab32672), and rabbit anti GluK1 (1:500, Abcam, ab67316). The slices were washed with PBST and incubated with the secondary antibodies, anti-chicken-FITC (Sigma; F8888) and anti-mouse-Alexa 594 (Molecular Probes; A11032), anti-mouse-Alexa 488 (Molecular probes; A11019), and anti-rabbit-Alexa 488 (Molecular Probes; A21206), and DAPI (nuclear marker). The slices were mount with mowiol-NPG and the pictures were taken with the confocal microscope Leica SPEII.

### Western blot

The proteins were extracted from fresh brain tissues (ACC and RSC) from postnatal 16 mice after decapitation. The extraction was made with RIPA buffer (Millipore; 20–188) in azote and sonicated later. The total amount of proteins was detected with Pierce BCA protein assay kit (Thermo Scientific; 23225). After denaturalization (at 92 ºC, 10 min with β-mercaptoethanol) 20 µg of proteins of each sample were loaded on SDS–polyacrylamide gel (12%) and electrophoresed followed by wet transfer in nitrocellulose blotting membrane (Amersham; 10600007). The membranes were incubated with primary antibodies overnight at 4 ºC (with TBST and BSA 5%): rabbit GluA2 (1:4000, Fisher; AB1768IMI), mouse anti-GluA1 (1:1000, Rockland, 200-301-D61), rabbit anti-GluK5 (1:1000, Abcam, ab32672), and mouse GAPDH (1:10,000, GeneTex; GTX627408). The membranes were washed with TBST and incubated with the secondary antibodies conjugated with HRP: anti-rabbit (1:4000, Vector; BA-1000) and anti-mouse (1:4000, Vector; BA-9200). The membranes were treated with Immobilon Forte (Millipore; WBLU0100) and exposed in dark room. Images were taken with the Amersham Bioimager 680 and to analyze the ratio of gray values between samples Image J was used. The protein level was expressed in arbitrary units (A.U.) calculated as the quotient between the area of the GluA2 band over the area of the GAPDH band.

### Statistics

Data are given as mean ± SEM and the number of cases. Statistical analysis was made with SigmaStat3.2 (SystatSoftwareInc., USA). Comparisons were made with the Student’s *t* test or the Mann–Whitney Rank Sum test (when sample values did not follow a normal distribution as checked with the Kolmogorov–Smirnov test). For percentage comparison in Fig. 3C, chi-squared (*χ*^2^) test was used. Statistical significance values were depicted using the following code: (*) *p* value < 0.05; (**) *p* value < 0.01; (***)* p* value < 0.001.

## Results

### Cortical networks of *Lis1/sLis1* mice do not display enhanced excitability

**Fig. 1:**
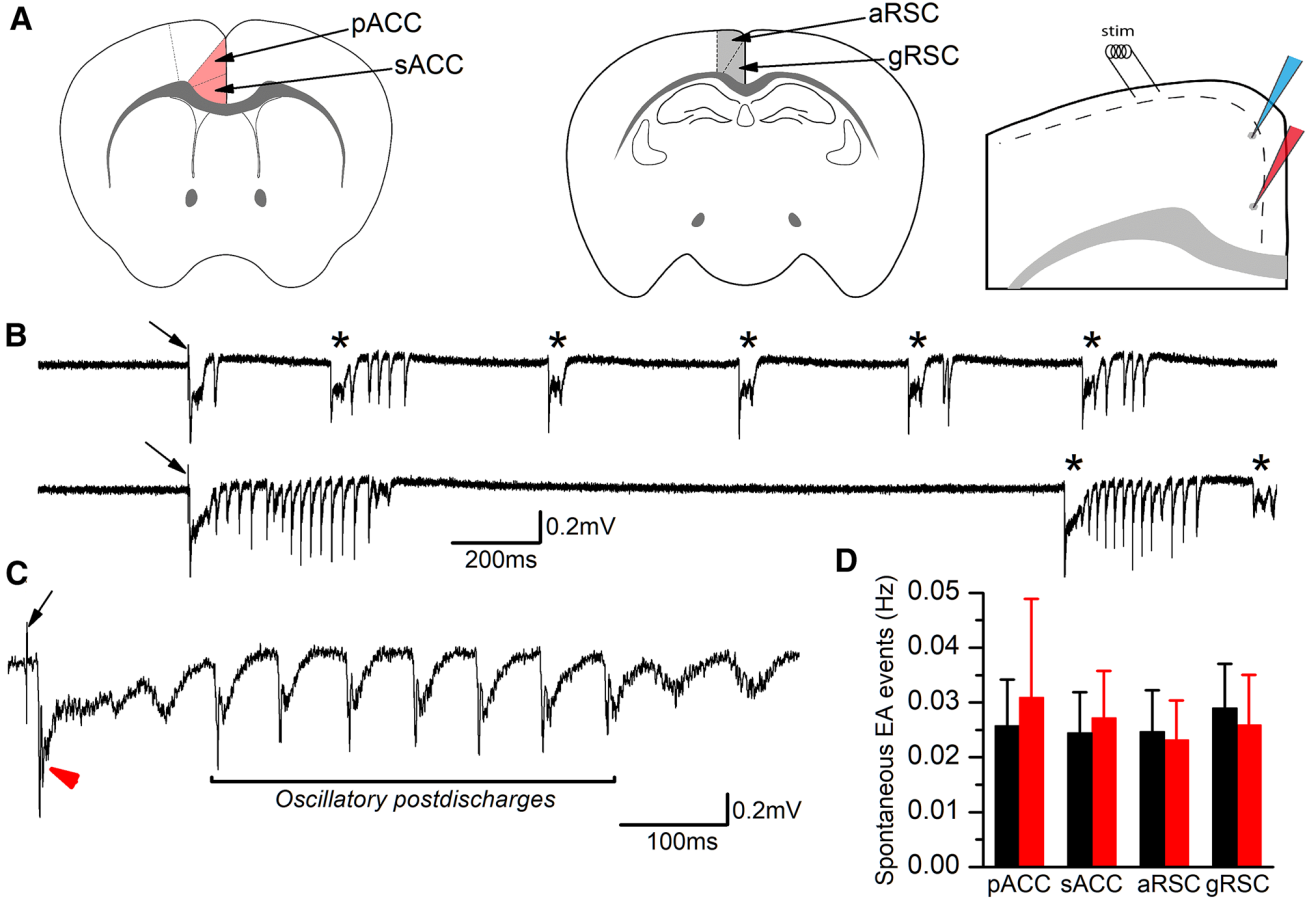
Epileptiform activity in the cingulate cortex. **A** Drawings of coronal sections of the mouse brain showing the anterior and posterior parts of the cingulate cortex. The anterior part (left panel) corresponds to the anterior cingulate cortex (ACC, shaded area), and the posterior part (right panel) is the retrosplenial cortex (RSC, shaded area). Right panel: arrangement of the stimulus electrode (“stim”) and the extracellular recording electrodes in both cortical areas. The dotted line shows the approximate limit between layer I and layer 2/3. The recording electrodes show typical recording positions in the more dorsal areas (pACC or gRSC, blue electrode) or in the more ventral areas (sACC or gRSC, red electrode). **B** Representative examples of epileptiform discharges recorded from a pACC slice in response to the stimulation of layer 1 (arrows: stimulus artifact). Asterisks mark spontaneous discharges. **C** Example of an evoked EA discharge shown at enlarged time scale; the arrow marks the stimulus artifact, the double red arrowhead marks the initial negative peak, and the horizontal bracket marks the period of oscillatory post-discharges. Recording obtained from a different pACC slice. **D** Frequency of spontaneous discharges measured in each one of the cortical areas studied (black bars: slices from WT animals; red bars: slices from *Lis1/sLis1* animals). The frequency was calculated as the average over a period of 10 min. of continuous recording

To investigate the consequences on cortical network activity of LIS1 dysfunction caused by LisH domain deletion in heterozygosis, we analyzed the properties and the propagation along layer 2/3 of epileptiform discharges evoked by electrical stimuli in conditions of enhanced cortical excitability. To do this, electrophysiological extracellular recordings were performed in brain slices from postnatal juvenile (P14–P16) mice, either mutant (*Lis1/sLis1)* or wild type (WT) littermates. Recordings were obtained under conditions of enhanced excitability (bicuculline 10 µM, 5 mM extracellular K^+^, and 1.2 mM Ca^++^; see “Methods”). Following our previous study on epileptiform activity propagation (Rovira and Geijo-Barrientos 2016), we recorded from two different cingulate cortical regions, the anterior cingulate cortex (ACC) and the retrosplenial cortex (RSC; Fig. 1A); these two areas were subdivided further in primary and secondary ACC (pACC and sACC) and dorsal agranular and granular RSC (aRSC and gRSC). In each slice, a stimulus electrode was placed on cortical layer 1 and recordings with an extracellular recording electrodes were performed targeting different positions along the cortical layer 2/3 (see scheme of the experimental design in Fig. 1A, right panel). Across experiments, stimulus parameters were stable, with minor adjustments of intensity (100–200 µA, 0.1 ms; see “Methods” for details on stimulus intensity adjustment). In these conditions, single-pulse electrical stimuli (100–200 µA, applied at 0.033 Hz) reliably induced stereotyped responses similar to those associated to seizure events recorded in similar conditions, which are the outcome of the coordinated firing of a local population of neurons (Rovira and Geijo-Barrientos 2016). We refer to this type of activity as epileptiform activity (EA). Figure 1B shows two representative extracellular recordings containing EA events. EA events were characterized by an initial negative peak after a fixed latency from the stimulus artifact (indicated, respectively, by an asterisk and an arrow in Fig. 1C), which was often followed by a variable number of post-discharges of smaller amplitude and shorter duration (indicated by a bracket in Fig. 1C); we refer to these events as “evoked EA events”. The post-discharges following the initial negative peak can persist for as long as one second after the stimulus. In some slices, EA events not related to electrical stimuli were also observed during the long inter-stimulus period (30 s; Fig. 1B); we refer to these events as “spontaneous EA events”.

**Fig. 2:**
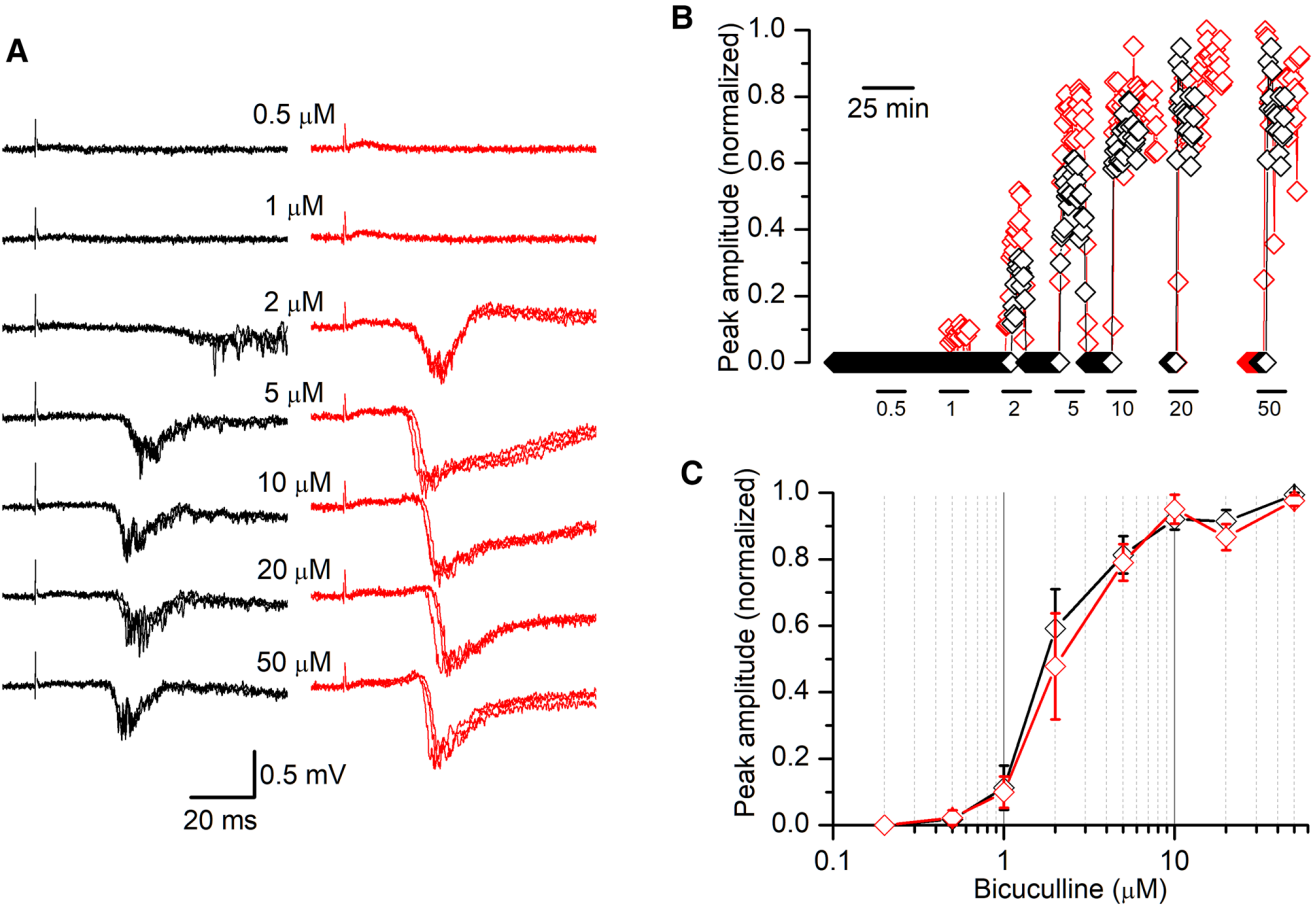
Dose–response relationship of the effect of bicuculline on epileptiform discharges. **A** examples from a WT slice (left, black traces) and a *Lis1/sLis1* slice (right, red traces) of the epileptiform discharges evoked in the presence on increasing concentrations of bicuculline. Three consecutive responses superimposed in each panel; stimulus artifacts blanked (0.5 ms). **B** Time course of the effect of bicuculline in the slices shown in panel A. The bars on the bottom show the times of successive applications of bicuculline at concentrations from 0.5 to 50 µM; between the application of 10 and 20 µM and between 20 and 50 µM there were long periods of bicuculline washout (not shown in the figure). **C** Dose–response relationship of the effect of bicuculline in the slices in which a complete series of bicuculline concentrations were applied (from 0.2 to 50 µM; WT and *Lis1/sLis1* *n* = 4)

The presence of spontaneous activity is directly related to the enhanced excitation level of the underlying neuronal network caused by our experimental conditions. Thus, we decided to quantify the frequency of these spontaneous events in the *Lis1/sLis1* mouse cortex to assess whether its cortex was more excitable. The frequency of spontaneous EA events was determined as the average during prolonged continuous recordings (10 min). Interestingly, the frequency of spontaneous EA events was similar in both genotypes (Fig. 1D). To analyze the sensitivity of the cortical circuits to the generation of EA we studied the responses to increasing concentrations of bicuculline, from 0.2 to 50 µM (Fig. 2). In the pACC, the threshold for the onset of EA was about 0.5 µM in both WT and *Lis1/sLis1* slices, and the maximum size of the epileptiform discharges was obtained with concentrations above 10 µM; the half-maximal effective concentration (EC_50_) of bicuculline was 1.99 ± 0.29 µM (WT slices, *n* = 4) and 2.24 ± 0.62 µM (*Lis1/sLis1* slices, *n* = 4). In the aRSC, the values of EC_50_ were also similar in WT and *Lis1/sLis1* slices (WT slices: 2.25 ± 0.46 µM, *n* = 2; *Lis1/sLis1* slices: 2.36 ± 0.39 µM, *n* = 3). The EC_50_ was calculated from the fit of a Hill equation to the data of those slices in which a complete series of concentrations of bicuculline (from 0.2 to 50 µM) were tested, and the difference between WT and *Lis1/sLis1* was not statistically significant in both ACC and RSC areas. Note that, both in WT and *Lis1/sLis1* slices, there were no epileptiform discharges at concentrations of bicuculline lower than 0.5 µM. This lack of differences in the dose–response relationship of the effect of bicuculline indicates that LisH domain deletion in heterozygosis has no impact on the overall cortical network excitability.

### The frequency of the oscillatory post-discharges is altered in the *Lis1/sLis1* cortex

**Fig. 3:**
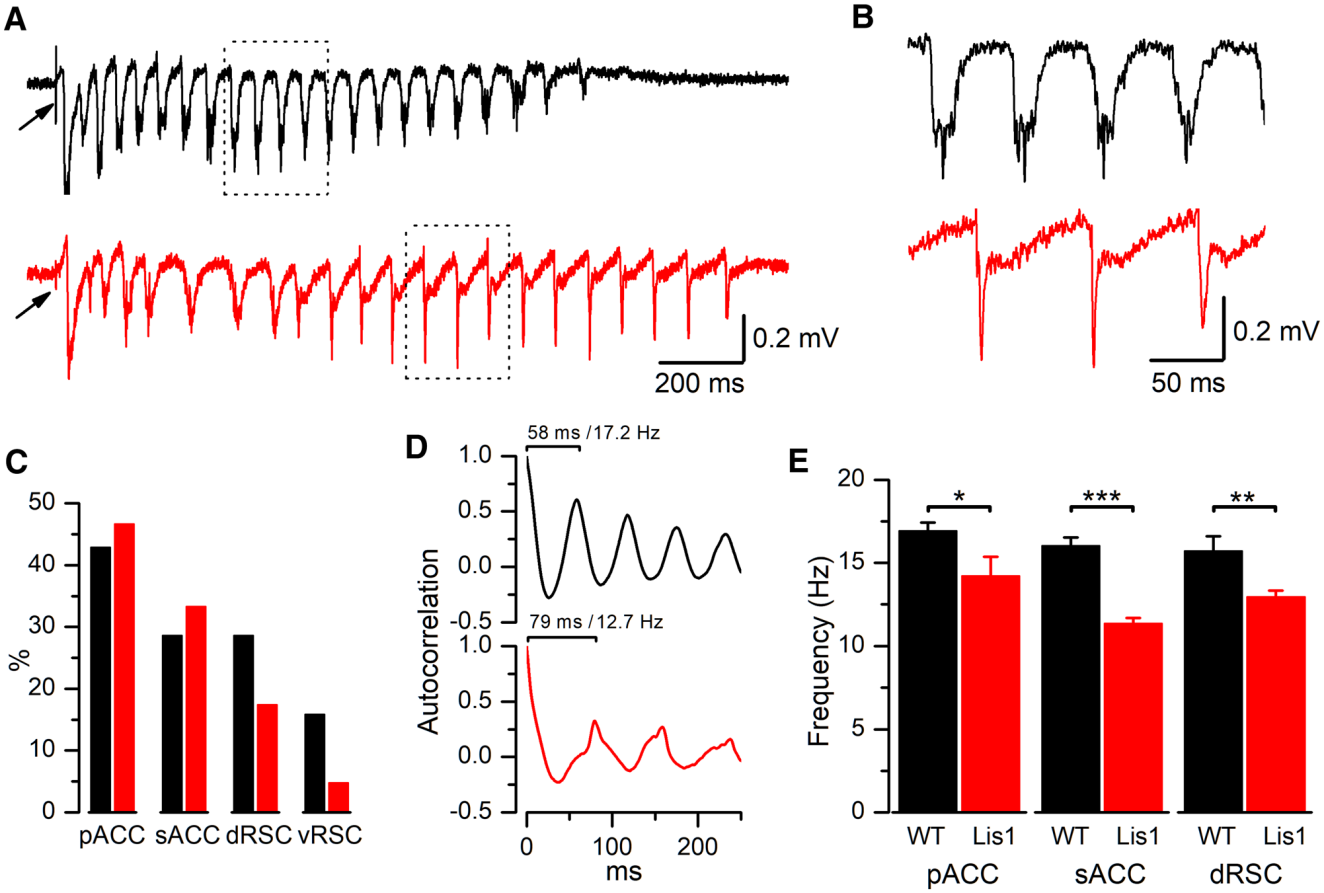
Frequency of the oscillatory post-discharges. **A** Representative recordings of EA from pACC, showing the initial negative peak following the stimulus artifact (arrows) and the associated oscillatory post-discharges (black trace is from a WT animal and red trace is from a *Lis1/sLis1*). **B** Detail of the segments of the recordings showed in (**A**) presented at a larger scale to show the higher frequency in WT slices. **C** Percentage of slices presenting epileptiform activity with post-discharges; number of slices (WT, *Lis1/sLis1*): pACC = 21, 15; sACC = 21, 15; aRSC = 21, 23; gRSC = 19, 21. **D** Autocorrelation plot of the traces shown in panel (**A**); it is clear the presence of regular oscillatory activity. **E** Frequency of oscillatory post-discharges; number of slices (WT, *Lis1/sLis1*): pACC = 7, 5; sACC = 5, 5; aRSC = 5, 7. In the gRSC, it was not possible to measure the frequency of the oscillatory post-discharges due to their infrequent presence

In some slices, we observed the emergence of prolonged oscillatory post-discharges following the initial negative component of the EA. These oscillatory post-discharges are illustrated in the examples shown in Figs. 1C and 3A, B, and they were apparent in both evoked and spontaneous EA events. The emergence and oscillation frequency of these post-discharges is determined by local recurrent excitatory glutamatergic connections established among neighbor pyramidal neurons (Castro-Alamancos and Rigas 2002) and, therefore, the analysis of these post-discharges could reveal the presence of abnormalities in the *Lis1/sLis1* cortical circuits. The results of this analysis are shown in Fig. 3. Figure 3A, B shows representative examples of evoked oscillatory post-discharges recorded from WT and *Lis1/sLis1* slices. Typically, post-discharges appeared as relatively regular oscillations (see the prominent peaks in the autocorrelogram shown in Fig. 3D) that followed the initial negative peak and that lasted for several hundreds of ms. First, we quantified the percentage of slices from either WT or *Lis1/sLis1* cortex presenting these oscillatory post-discharges to evaluate the emergence of this activity. The percentage of slices from *Lis1/sLis1* or WT mice showing oscillatory post-discharges was not significantly different (Fig. 3C; comparisons made with the chi-squared test). The presence of oscillatory post-discharges was particularly scarce in the gRSC. Then, we compared the frequency of these oscillatory post-discharges (Fig. 3E). In slices from WT animals, the oscillation frequency was similar across cortical regions and comparable to previously published results under similar experimental conditions (Castro-Alamancos and Rigas 2002; Rovira and Geijo-Barrientos 2016). In the ACC (pAAC: WT 16.91 ± 0.50 Hz *n* = 7 vs *Lis1/sLis1* 14.2 ± 1.17 Hz, *n* = 5; *p* = 0.038, Student’s *t* test. sAAC: WT 16.02 ± 0.49 Hz *n* = 5 vs *Lis1/sLis1* 11.36 ± 0.33 Hz, *n* = 5; *p* < 0.001, Student’s *t* test) and in the aRSC (WT 15.71 ± 0.88 Hz *n* = 5 vs *Lis1/sLis1* 12.95 ± 0.37 Hz, *n* = 7; *p* = 0.009, Student’s *t* test) the oscillation frequency was smaller in the *Lis1/sLis1* cortex (Fig. 3E). In the gRSC, the oscillation frequency could not be measured because in this area the oscillatory post-discharges were scarce and very irregular. However, while the frequency of the oscillatory post-discharges was lower in the *Lis1/sLis1* cortex, their total duration was similar to WT cortex; in the pACC the duration was: WT 1.34 ± 0.11 s (*n* = 6), *Lis1/sLis1* 1.64 ± 0.19 s (*n *= 6); in the sACC was: WT 1.65 ± 0.77 s (*n* = 6), *Lis1/sLis1* 1.75 ± 0.09 s (*n* = 5); and in the aRSC was: WT 1.19 ± 0.03 s (*n* = 5), *Lis1/sLis1* 0.83 ± 0.17 s (*n* = 6); All differences were not significant. This oscillatory component of the EA is caused by complex mechanisms involving synaptic mechanisms and intrinsic electrophysiological properties of the interconnected neurons (Avoli et al. 2002; De Curtis et al. 2012). This makes difficult to derive more concrete conclusions about the mechanisms underlying these findings of a lower frequency and a similar duration of the post-discharges.

### Epileptiform activity propagates more slowly in *Lis1/sLis1* cortex

**Fig. 4:**
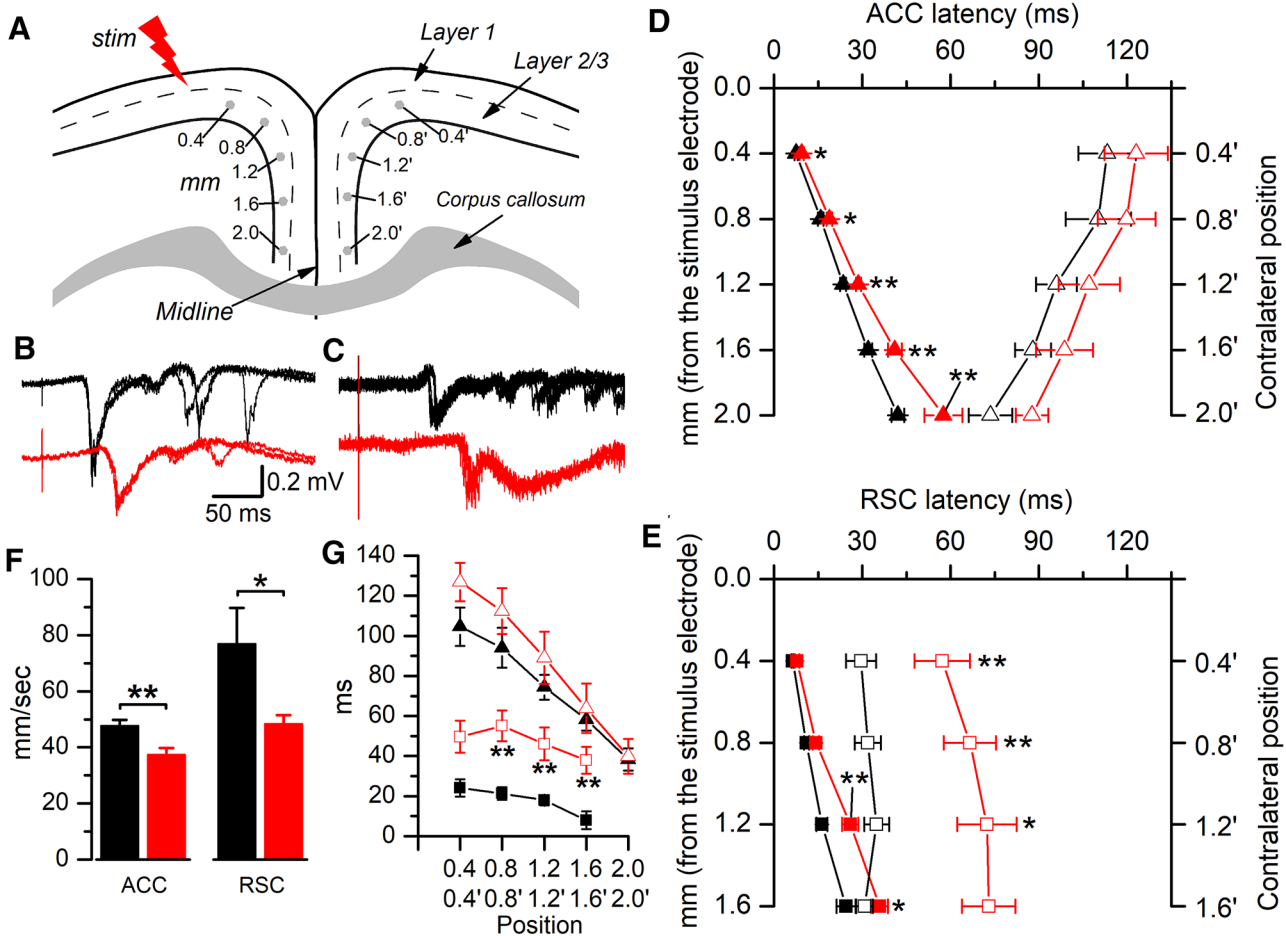
Propagation of epileptiform activity along layer 2/3. **A** Drawing of a coronal slice of the cingulate cortex showing the arrangement of the stimulus electrode (“*stim*”) and the recording sites in layer 2/3 used to measure the propagation of epileptiform activity; this drawing is modified from Robles et al. (2020). In the ipsilateral hemisphere (respect to the stimulus electrode) recording sites were placed at 0.4 mm intervals from the stimulus electrode to cover the whole layer 2/3 of the cingulate cortex; in the contralateral hemisphere recording sites were homotopic in respect to the ipsilateral side, and are marked as 0.4´–2.0´. **B**, **C** Representative recordings from the ipsilateral ACC (panel **B**; recording site 1.6 mm) and from the contralateral ACC (panel **C**; recording site 1.6´). Three consecutive responses shown overlapped in each panel. Scale bars in (**B**) apply to (**A**). In all panels of this figure black traces and symbols are from WT animals and red traces and symbols are from *Lis1/sLis1*. **D** Onset latencies of the responses recorded in the ipsilateral ACC (filled triangles) and in the contralateral ACC (open triangles). **E** Onset latencies of the responses recorded in the ipsilateral RSC (filled triangles) and in the contralateral RSC (open triangles); in the RSC we only explored up to 1.6 mm from the stimulus electrode due to the smaller size of the RSC. **F** Propagation velocity of epileptiform discharges along the ipsilateral layer 2/3; the propagation velocity was measured from the linear fit of the latencies of the epileptiform discharges recorded in the different sites of the ipsilateral hemisphere. **G** Time interval between recordings obtained from homotopic sites in the ACC (triangles) and in the RSC (squares). In all panels the asterisks placed near the red symbols show the significance of the comparison with the homotopic ipsilateral site. Recordings and values are presented in black for wild type and red for *Lis1/sLis1* cortex (ACC: WT = 25 slices, *Lis1/sLis1* = 15 slices; RSC: WT = 11 slices, *Lis1/sLis1* = 21 slices)

EA evoked by electrical stimuli is not restricted to the vicinity of the stimulus electrode, but can propagate through the cortex; this propagation may also reach the contralateral hemisphere (relative to the stimulus electrode) when the callosal connectivity between both areas is intact (Walker et al. 2012; Rovira and Geijo-Barrientos 2016). EA propagation along layer 2/3 was studied and compared between WT and *Lis1/sLis1* ACC or RSC using a procedure previously described (Rovira and Geijo-Barrientos 2016) Briefly, this procedure consisted in the extracellular recording of EA evoked by electrical stimuli applied to layer 1 with an extracellular electrode successively placed at 0.4 mm intervals from the stimulus electrode (Fig. 4A). Also, the recording electrode was placed at homotopic sites in the contralateral hemisphere (marked as 0.4´–2.0´ in Fig. 4A) to study the propagation to the contralateral hemisphere. Figures 4B, C shows examples of EA recorded in the ipsi- and contralateral hemispheres. Figures 4D, E shows the latencies of the EA recorded at the different recording sites in the ACC (Fig. 4D) and RSC (Fig. 4E) in WT and *LIS1/sLis1*. In the ipsilateral hemisphere, the longer latencies of the responses recorded at increasing distances from the stimulus represent the propagation of evoked responses from the stimulation; the velocity of this propagation was calculated in each slice from the linear fit of the latency values.

In the ipsilateral side of ACC and RSC the latencies were longer in *Lis1/sLis1* slices (Fig. 4D, E) indicating that the velocity of propagation of EA was lower in both cortical areas of *Lis1/sLis1* slices (Fig. 4F) These propagation velocities were: in the ACC WT 47.69 ± 2.16 mm/s (*n* = 25), *Lis1/sLis1* 37.34 ± 2.43 mm/s (*n* = 15), *p* = 0.004; in the RSC WT 76.83 ± 12.89 mm/s (*n* = 12), *Lis1/sLis1* 49.16 ± 3.92 mm/s (*n* = 23), *p* = 0.011. The latencies and propagation velocities found in WT slices were comparable to those previously described in a different mouse strain (Rovira and Geijo-Barrientos 2016); in WT ACC the velocity of propagation was slower than in WT RSC (*p* = 0.043, Mann–Whitney rank sum test). In *Lis1/sLis1* cortex the velocity of propagation was also slower in the ACC than in the RSC (37.34 ± 2.43 mm/ms, *n* = 15, vs 48.33 ± 3.12 mm/ms, *n* = 21, respectively; *p* = 0.009, Mann–Whitney rank sum test); however, the difference between ACC and RSC was larger in WT tan in *Lis1/sLis1* cortex (161% vs 129% change from ACC to RSC, respectively); this fact is probably a consequence of the different organization of the neuronal local circuits of layer 2/3 of the ACC and RSC, and suggests an heterogeneous impact of *LIS1* dysfunction along cingulate cortical areas.

In the contralateral hemisphere, the latencies were longer than in the ipsilateral hemisphere; the contralateral latencies, however, represent probably the building-up of the EA in the contralateral layer 2/3 and not the interhemispheric conduction time of the callosal axons, which is shorter (about 8 ms in the aRSC, Sempere-Ferrandez et al. 2018). In the ACC there were no differences between WT and *Lis1/sLis1*, and in the RSC the contralateral latencies were longer in *Lis1/sLis1* slices (Fig. 4D, E). The interhemispheric time interval was measured as the latency different between homotopic recording sites (Fig. 4G); This time interval was longer in *Lis1/sLis1* slices, but only in the RSC, indicating again the different consequences of *LIS1* dysfunction in both cortical areas.

The above data about the propagation of EA clearly indicate the presence of alterations in the structure and/or the function of the cortical circuits of layer 2/3 in *Lis1/sLis1* cortex. These data show also that the consequences of LIS1 mutation differ depending on the cortical area. Several mechanisms may be implicated in these abnormalities, such as changes in the intrinsic properties of cortical neurons or changes in the structure of the neuronal circuits; The propagation of EA in the neocortex is mainly supported by excitatory synapses (Pinto et al. 2005) hence alterations in glutamatergic transmission may also be implicated.

### Regular spiking pyramidal neurons of *Lis1/sLis1* mice display normal electrophysiological properties, but show altered sEPSCs

**Fig. 5:**
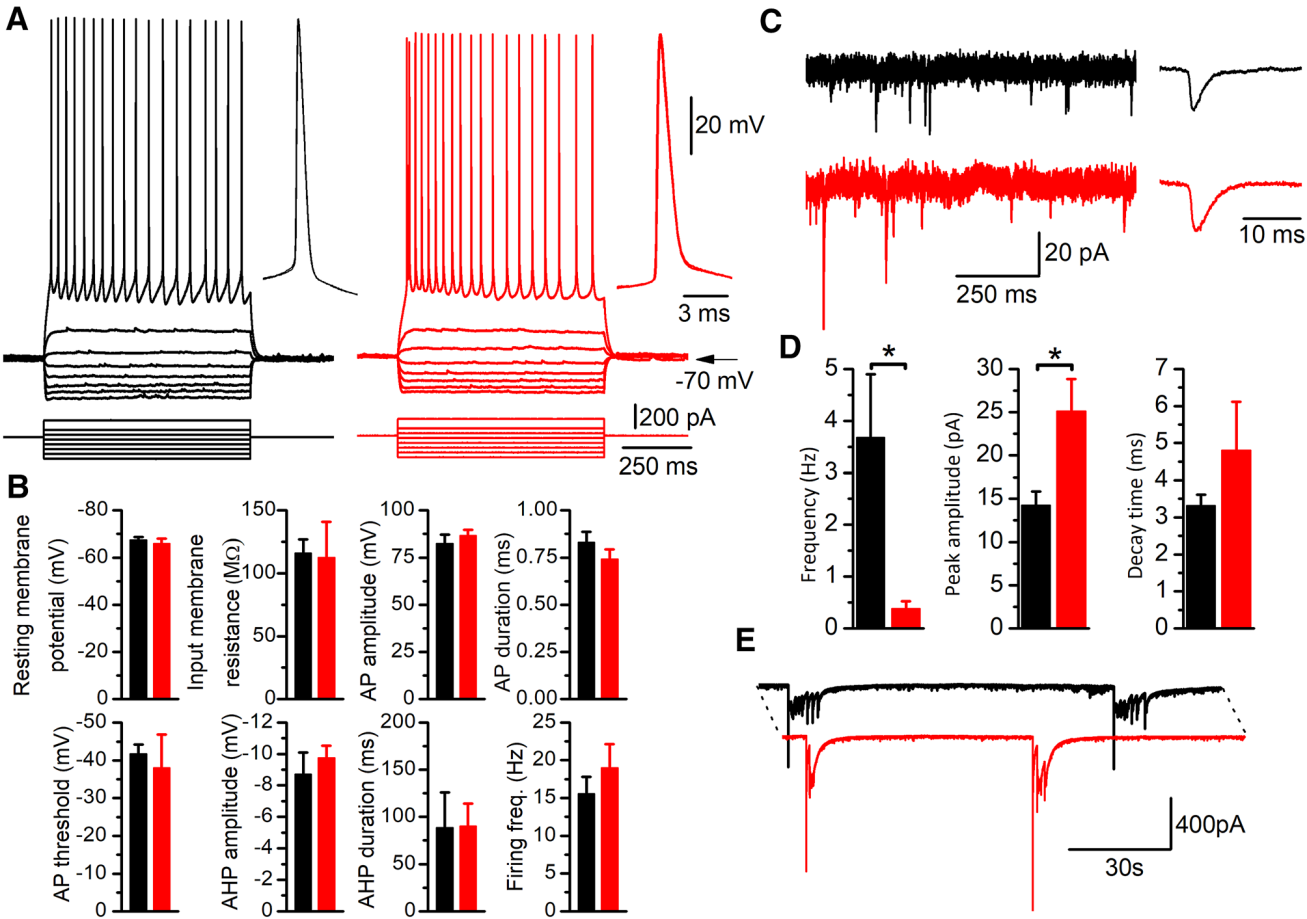
Electrophysiological properties and sEPSC in layer 2/3 regular spiking pyramidal neurons. **A** Examples of the responses to hyper- and depolarizing current pulses of a regular spiking pyramidal neuron from a WT animal (black traces) and from a *Lis1/sLis1* animal (red traces). The inset show two action potentials (the 5th and the 6th of the train) superimposed at a larger time scale; the dotted lines show the threshold level. **B** Electrophysiological parameters measured in WT (black) and *Lis1/sLis1* (red) pyramidal neurons. The resting membrane potential was measured immediately after entering into the whole-cell mode; the membrane input resistance was measured from the responses to small hyperpolarizing current pulses; the threshold was measured at the 1st action potential in a just-threshold response; the action potential peak amplitude was measured from the threshold and the duration was measured at half amplitude; the peak amplitude of the action potential after-hyperpolarization (AHP) was measured from the threshold level, and the AHP duration was measured from the peak to 50% of the peak amplitude. **C** Left panel examples of membrane current recorded at − 70 mV in a WT neuron (black) and a *Lis17sLis1* neuron (red); the right panel shows the averaged sEPSC detected in a 30 s recording interval. **D** Frequency of sEPSCs and values of peak amplitude and time constant of the decay phase of the averages sEPSCs detected in a 30 s interval (WT: 8 neurons; *Lis1/sLis1*: 13 neurons). **E** Long, continuous recording of membrane current at − 70 mV showing the large bursts of excitatory currents caused by spontaneous epileptiform discharges in a neuron from a WT slice (black trace) and a neuron from a *Lis1/sLis1* slice (red trace); dotted lines indicate that the time interval shown was the same for both recordings

To test whether the above results could be caused by alterations of neuronal electrophysiological properties we performed whole-cell recordings of layer 2/3 pyramidal neurons. Intracellular recordings were performed in gRSC, given that it shows a more acute phenotype than rostral ACC cortex. In layer 2/3 of this cortical area there are both regular spiking and late spiking pyramidal neurons (Kurotani et al. 2013; Robles et al. 2020); we only recorded and studied regular spiking pyramidal neurons because the generation of epileptiform activity in this cortical area is restricted to this type of pyramidal neurons (Robles et al. 2020). Figure 5A shows representative recordings of regular spiking pyramidal neurons from WT and *Lis1/sLis1* gRSC. The electrophysiological properties were similar across genotypes (Fig. 5B); note that the shorter action potential duration observed in regular spiking pyramidal neurons from *Lis1/sLis1* parietal cortex at postnatal day 30 (Valdés-Sánchez et al. 2007) was not apparent in our data; this could be to the earlier postnatal development stage of our experiments (P14-16 respect to P30 in Valdés-Sánchez et al. 2007) or to the presence of differences among different cortical areas (RSC in our experiments vs parietal cortex in Valdés-Sánchez et al. 2007). Overall, the firing rate in response to step-current injections, the resting membrane potential, and the threshold fort spike firing remained unaltered indicating that LIS1 dysfunction did not affect the intrinsic excitability of pyramidal neurons (Fig. 5B).

In these recording conditions, spontaneous excitatory synaptic currents (sEPSC) were easily detected (Fig. 5C). The quantification of sEPSC may give information about the excitatory input received by pyramidal neurons. In pyramidal neurons from *Lis1/sLis1* animals the frequency of sEPSCs was lower, and their peak amplitude was larger than in WT neurons (Fig. 5C, D). The sEPSCs were detected and measured in periods of 30 s selected just before the firing of a large burst of synaptic activity caused by an epileptiform discharge. The different sEPSC frequency may be caused by a prolonged firing of some neurons after the epileptiform discharges; in the neurons recorded intracellularly we did not detect this type of tonic firing, but we cannot exclude the presence of a few neurons that maintain a continuous firing in the inter EA periods in the *Lis1/sLis1* cortex; in the WT cortex the frequency of sEPSCs was lower, but again, we cannot determine whether this difference was due to lower number of firing neurons, to a lower firing frequency or both. On the other side, the different amplitude of the sEPSCs could be related to changes in the postsynaptic receptors (see below the data from experiments using AMPA receptor blockers) or to the transmitter release mechanisms.

The experiments of sEPSC recording were based on continuous prolonged recording of the membrane current at a holding potential of − 70 mV. In these recordings, it was apparent the presence of large barrages of excitatory currents caused by epileptiform activity (Fig. 5E); the frequency of these bursts of excitatory currents was similar in WT and *Lis1/sLis1* animals (0.018 ± 0.0057, *n* = 8 and 0.022 ± 0.007, *n* = 13, respectively), which is consistent with our finding of similar frequency of spontaneous epileptiform activity events recorded with extracellular electrodes (Fig. 1D).

### Glutamate receptor alterations in *Lis1/sLis1* cingulate cortex

**Fig. 6:**
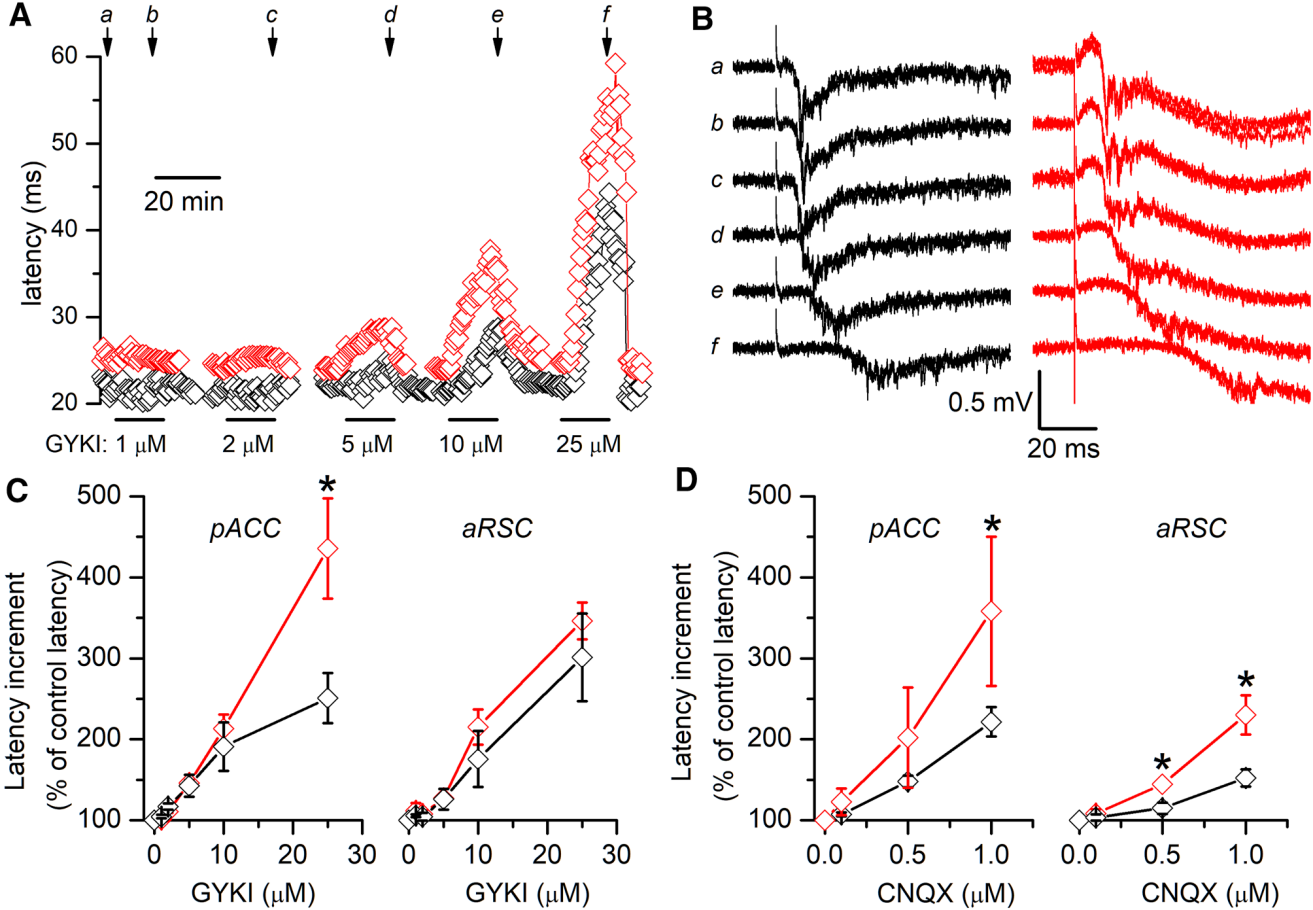
Latency increment induced by GYKI 53,655 and CNQX. **A**, **B** Representative examples of the effect of increasing concentrations of GYKI53655 on the latency of the epileptiform discharges evoked in two aAAC slices (WT slice, black symbols and traces; *Lis1/sLis1* slice red symbols and traces). Panel **A** shows the time course of the latency during the application and washout of GYKI53655 and panel **B** shows examples of the discharges obtained in control (**a**) and at the end of the application of different concentrations of GYKI53655 from 1 to 25 µM (**b**–**f**, at the times shown in panel **A**. Three consecutive recordings shown superimposed in each panel; the central part (0.01) ms of the stimulus artifacts has been blanked in all recordings. **C** Latency increment induced by GYKI53655 in pACC slices. Comparisons made with the Mann–Whitney rank sun test; number of slices: pACC WT 6–7, *Lis1/sLis1* 4–6; dRSC WT 7, *Lis1/sLis1* 5–6. D, Latency increment induced by CNQX in aRSC. Comparisons made with the Mann–Whitney rank sun test; number of slices: ACC WT 11, *Lis1/sLis1* 8; RSC WT 7–10, *Lis1/sLis1* 5. In panels **C**, **D,** the latency increments are shown normalized respect to the latency value measured in control conditions (before the application of GYKI or CNQX); the control latency is shown as 100% and a particular latency increment is shown as a percentage with respect to the control value

**Fig. 7:**
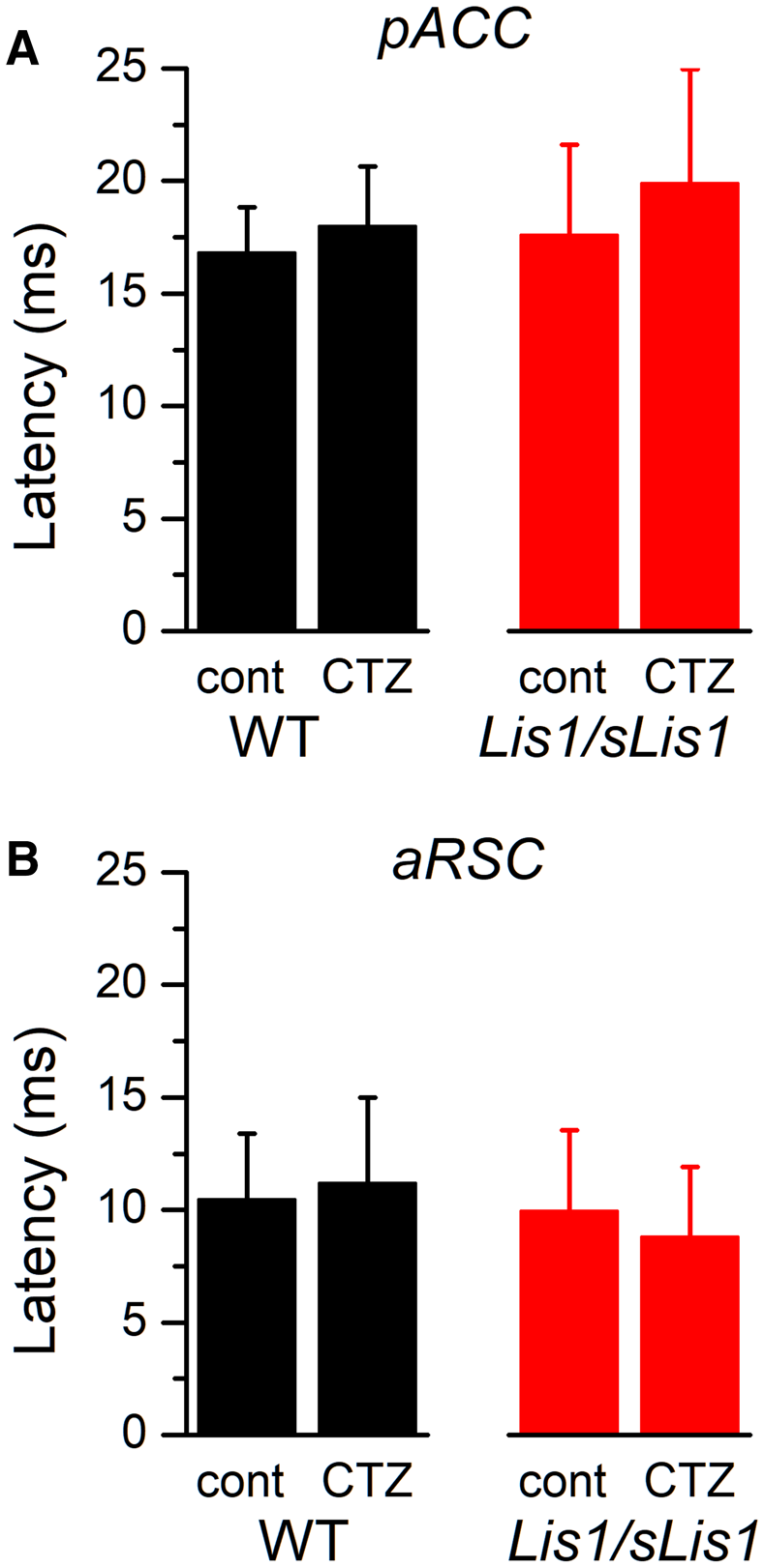
Effect of cyclothiazide on the latency of the EA. Latency of the EA recorded from WT (black columns) and *Lis1/sLis1* (red columns) slices in the pACC (panel **A**; WT *n* = 6 slices, *Lis1/sLis1* *n* = 5 slices) and in the aRSC (panel **B**; WT *n* = 5 slices, *Lis1/sLis1* *n* = 4 slices) in control conditions and after the application of 100 µM cyclothiazide (CTZ)

Ipsilateral and contralateral propagation of EA depends on excitatory glutamatergic transmission between pyramidal neurons highly reliant on AMPA type receptors (Alefeld et al. 1998; Pinto et al. 2005). This suggest that, among other possible mechanisms, the slower propagation velocity of EA observed in *Lis1/sLis1* slices could be caused by abnormalities in AMPA type glutamate receptors. To check for alterations of AMPA receptors in *Lis1/sLis1* animals we studied the effect on EA propagation of several drugs acting on AMPA receptors: a selective AMPA receptor blocker (GYKI53655), an AMPA/kainate receptor blocker (CNQX), and a positive modulator of AMPA receptors (cyclothiazide). To do these experiments we recorded extracellularly in the ipsilateral hemisphere of the pAAC and the aRSC, at approximately 0.8 mm from the stimulus electrode. In the pACC and the aRSC from WT slices, bath application of GYKI53655 resulted in a reversible, dose-dependent increase in the latency of epileptiform discharges (Fig. 6). In the pACC this latency increment was significantly larger in *Lis1/sLis1* slices than in WT slices, but in the aRSC the latency increment was similar in both genotypes (Fig. 6C). On the other hand, the application of CNQX also induced an increase in latency in both pACC and aRSC, but in both cortical areas the latency increase was larger in the *Lis1/sLis1* cortex than in the WT cortex (Fig. 6D). In fact, in the aRSC area the increase of latency induced by CNQX was clearly larger and apparent at lower concentrations than the increase induced in pACC. Since the specific blocking action of GYKI53655 on AMPA receptors has been used to disclose effects caused by kainate receptors (Paternain et al. 1995), these results point to the possibility that abnormalities in different types of receptors could be implicated in the differences observed in the propagation of EA in both cortical areas of *Lis1/sLis1* animals (see “Discussion”). We next tested the effect of cyclothiazide (CTZ, applied at 100 µM), a positive modulator of AMPA receptors; neither in the pACC nor in the aRSC CTZ produced a change in latency, either in WT slices or in *Lis1/sLis1* slices (Fig. 7).

**Fig. 8:**
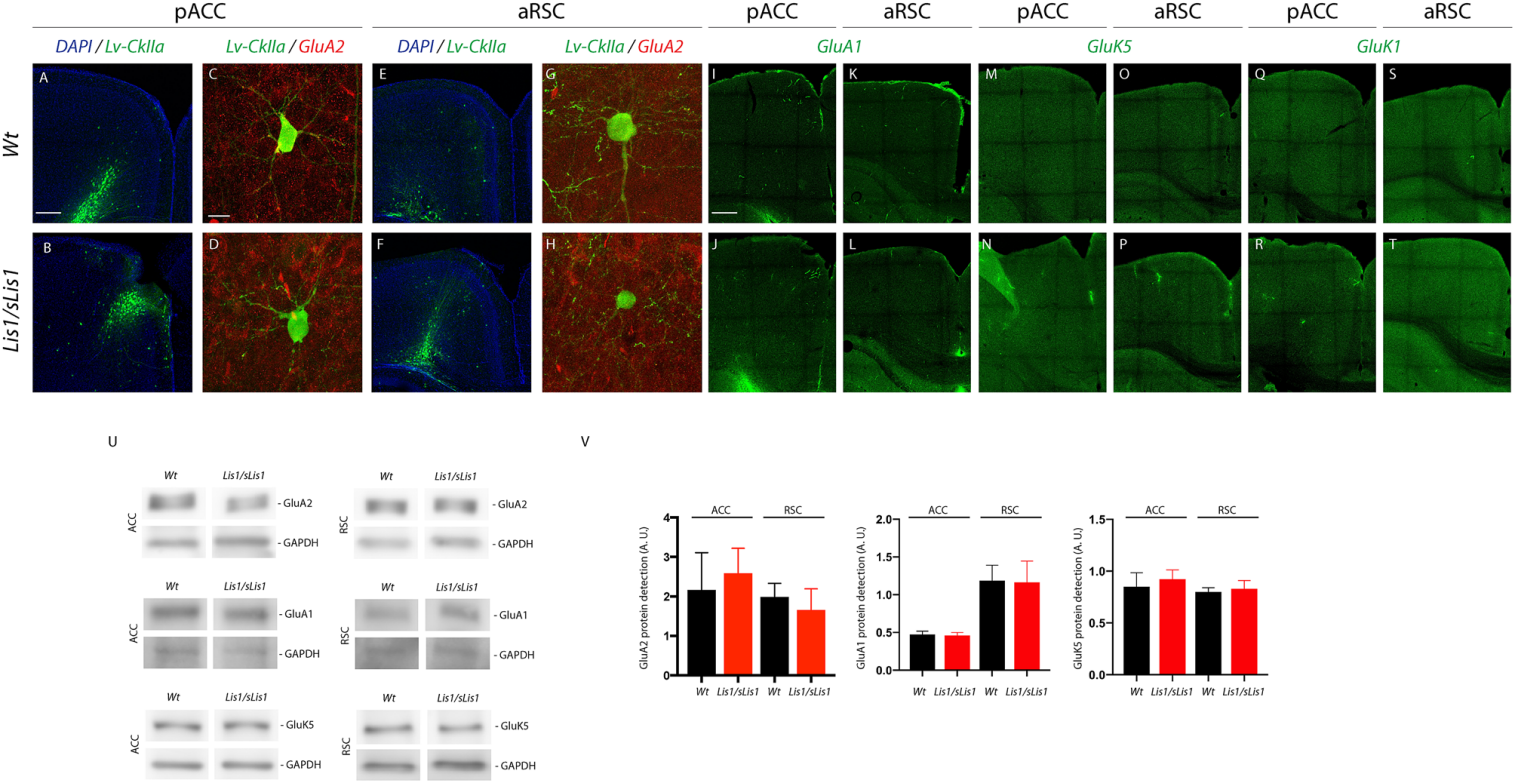
GluA2, GluA1, GluK5, and GluK1 subunit expression in the pACC and aRSC. **A**, **B** Lentiviral injection of LV-CkIIa-GFP (green) in ACC in *WT* and *Lis1/sLis1*. **B**, **D** GFP expression in pyramidal cells infected with LV-CkIIa-GFP (green) and GluA2 expression (red) in ACC in *WT* and *Lis1/sLis1*. **E**, **F** Lentiviral injection of LV-CkIIa-GFP (green) in RSC in *WT* and *Lis1/sLis1*. **G**, **H** GFP expression in pyramidal cells infected with LV-CkIIa-GFP (green) and GluA2 expression (red) in RSC in *WT* and *Lis1/sLis1*. **I**–**L** GluA1 expression (green) in ACC and RSC in *WT* and *Lis1/sLis1*. **M**–**P** GluK5 expression (green) in ACC and RSC in *WT* and *Lis1/sLis1*. **Q**–**T** GluK1 expression (green) in ACC and RSC in *WT* and *Lis1/sLis1*. **U** detection of total GluA2, GluA1, and GluK5 proteins in ACC and RSC cortices in *WT* and *Lis1/sLis1*. GAPDH is used as a control. **V** quantification of GluA2, GluA1, and GluK5 proteins in arbitrary units (AU); the western blot for GluK1 was not specific enough to be quantified. *T*-test analysis was not significant for all markers and for both cortices. GluA2-ACC: *WT* = 4, *Lis1/sLis1* = 5, *p* = 0.49; GluA2-RSC: *WT* = 4, *Lis1/sLis1* = 5, *p* = 0.49. GluA1-ACC: *WT* = 4, *Lis1/sLis1* = 5, *p* = 0.62; GluA1-RSC: *WT* = 4, *Lis1/sLis1* = 4, *p* = 0.91. GluK5-ACC: *WT* = 4, *Lis1/sLis1* = 4, *p* = 0.40; GluK5-RSC: *WT* = 3, *Lis1/sLis1* = 3, *p* = 0.59. Scale bars: in **A** 100 µm, in **B** 10 µm, and in I 100 µm

There are multiple possible abnormalities in the AMPA/kainate receptors of *Lis1/sLis1* cortex, and we checked the possibility of an altered number of AMPA receptors or an altered subunit composition of AMPA receptors by immunohistochemistry or western blot using an antibody against the GluA1 and GluA2 subunits of AMPA receptors and against the GluK1 and GluK5 subunits of kainate receptors. We explored the GluA2 subunit, given the critical role of this subunit in determining the calcium permeability and the mature properties of the AMPA receptors (Yuan and Bellone 2013). Both immunohistochemistry (to detect the distribution of these subunits) and western blot experiments (to detect the protein level) with a primary antibody against these subunits showed no significant differences between WT and *Lis1/sLis1* in pACC and aRSC cortical areas (Fig. 8). These results discard a change in the level of receptors containing this subunit as a possible cause of the electrophysiological alterations observed in the *Lis1/sLis1* cortex, but they leave a large number of possibilities open, among them the implication of other subunits or functional alterations in glutamate receptors.

## Discussion

LIS1 is a widely expressed protein involved in several cellular functions (Reiner and Sapir 2013). Since the isolation of *LIS1* gene (Reiner et al. 1993), a bunch of heterogeneous alterations along the encoding sequence have been described in lissencephalic patients (Saillour et al. 2009; Chong et al. 1997; Uyanik et al. 2007), ranged from point mutations to big deletions. Despite the poor knowledge about how *LIS1* dysfunction affects neuronal networks, it has been proposed that recurrent intractable epileptic crisis, a hallmark of lissencephaly, are the consequence of a deficit in the inhibitory control of these networks (Dobyns 2010).

In this work, we performed a set of experiments in the cingulate cortex of *Lis1/sLis1* mice, a model lacking the LisH domain in heterozygosis, to understand the impact of this genetic context in the properties of the neocortical networks implicated in the generation of epileptiform activity. The use of GABA_A_ blockers, such as bicuculline, to induce epileptiform electrical activity that propagates along the cortex has been described and studied for years, and the pharmacological disinhibition is a widely accepted model of epileptiform activity in the cerebral cortex (Chagnac-Amitai and Connors 1989; Pinto et al. 2005; see a review in Avoli et al. 2002). The use of bicuculline in our experiments allowed us to study epileptiform electrical activity in the *Lis1/sLis1* cortex, but it prevented us to study the contribution of the GABAergic transmission to the alterations that we describe here. Our finding that spontaneous epileptiform discharges appeared with similar frequency suggests that the excitability of cortical neural circuits was similar in *Lis1/sLis1* and WT cortices. This conclusion is reinforced by the finding of a similar EC_50_ of bicuculline for the generation of EA. However, we cannot discard the presence of subtle differences in excitability that were occluded by the partial blockage of GABA_A_ receptors by bicuculline used to evoke EA. Thus, our results indicate that the neuronal mechanisms controlling the emergence of epileptic activity are preserved and that the GABAergic mechanisms that prevent the apparition of EA are also preserved in the *Lis1/sLis1* cortex, since the same concentration of bicuculline produced the same frequency of EA events in WT and *Lis1/sLis1* cortex.

The oscillatory component of the EA had a lower frequency but the same overall duration in WT and *Lis1/sLis1* slices. This kind of oscillatory activity is caused by complex interactions between neuronal intrinsic electrophysiological properties and synaptic activity (Avoli et al. 2002; De Curtis et al. 2012). We show that some intrinsic properties of layer 2/3 pyramidal neurons that participate in the generation of oscillatory activity (membrane resting potential, membrane resistance, and action potential threshold) were similar in both genotypes; this discards the alterations of intrinsic neuronal properties as the cause of the lower frequency and suggests that its cause could be the presence of synaptic alterations, given that it has been described the modulation of this type of oscillatory post-discharges by AMPA receptors (Castro-Alamancos and Rigas 2002).

An important finding arise from the study of EA propagation along the cingulate cortex. Their propagation was slower, both in the anterior cingulate cortex and in the retrosplenial cortex; also, in the RSC the onset of EA in the contralateral hemisphere after its propagation through the corpus callosum took longer time. This finding clearly points to the presence of abnormalities in the cortical neuronal circuits supporting both the propagation of EA and its initiation in the neocortex, because the long latencies of the responses recorded in the contralateral cortex are due mostly to the process of EA building-up and not to the propagation through the corpus callosum, which takes much shorter times (Rovira and Geijo-Barrientos 2016; Sempere-Ferrández et al. 2018). The propagation of EA across neocortex in conditions of partial blocking of GABA_A_ receptors depends mostly on excitatory glutamatergic synapses (probably acting by AMPA receptors) connecting pyramidal neurons in the layer 2/3 (Pinto et al. 2005), but alterations in the intrinsic electrophysiological properties of the cortical pyramidal neurons could be also implicated in this slow propagation. Somatic whole-cell recordings showed that, in concordance with previous studies obtained from parietal cortex in the *Lis1/sLis1* model (Valdés-Sánchez et al. 2007), there were not alterations in the intrinsic properties related with the neuronal excitability, such as resting membrane potential, input membrane resistance, or action potential threshold. This was similar to the finding of absence of alterations of the intrinsic properties of hippocampal glutamatergic (Hunt et al. 2012) or GABAergic neurons (Jones and Baraban, 2007) in a mouse model lacking one allele of LIS1. Altogether, these findings suggest that LIS1 dysfunction does not have impact on the neuron electrophysiology despite its important role in different cellular processes.

We detected a reduced frequency and an increased amplitude of the sEPSCs recorded in pyramidal neurons of *Lis1/sLis1* cortex in comparison with pyramidal neurons from WT cortex. This finding is contrasting with the results by Greenwood et al. (2009), who showed, in a different *Lis1* mutant mouse (the heterozygous *Lis1*^*ex6neo−8*^), a large increase of the frequency of both spontaneous and miniature EPSC, but without changes in their peak amplitude; these abnormalities are probably due to an enhancement of glutamate transmission. In our model, the results with GYKI and CNQX suggest also the presence of abnormalities in the glutamate mediated synaptic transmission. We have not found alterations in the distribution or in the level of some glutamate receptor subunits (GluA1, GluA2, GluK1, and GluK5) studied with immunohistochemistry and western blot; this suggest that there are not structural differences in AMPA or kainate receptors related to these subunits, but other possibilities remain open, given the number of cellular processes in which the gen *LIS1* is involved. The lower sEPSC frequency could be caused by an altered overall network activity or by synaptic abnormalities (a decreased probability of transmitter release or synaptic failures); on the other side, the increased size of sEPSCs could be related to functional alterations of glutamate receptors. Since our experiments were done in the presence of bicuculline, with an enhanced neuronal excitability, the mechanisms underlying the decreased frequency of sEPSC could be due to alterations in transmitter release and/or in the functional properties of glutamate receptors.

Since the propagation of EA depends on excitatory glutamatergic transmission between pyramidal neurons that is highly dependent on AMPA type receptors (Alefeld et al. 1998; Pinto et al. 2005) we next hypothesized that the reduced velocity of propagation could be a consequence of alteration in the properties of these receptors. We tested this possibility by studying the modulation of the latency of EA discharges by drugs acting on AMPA / kainate receptors: GYKI 53,655, a selective blocker of AMPA receptors (Paternain et al. 1995), CNQX, a blocker of AMPA/kainate receptors and cyclothiazide, a positive modulator of AMPA receptors. Our findings suggest a different implication of AMPA and kainate receptors mediated transmission in the longer latencies observed in the pACC and aRSC of *Lis1/sLis1* animals. In the pACC both GYKI 53,655 and CNQX produced a larger increase in latency in *Lis1/sLis1* than in WT cortex; this indicates that abnormalities in synaptic transmission mediated by AMPA receptors would be sufficient to explain this result (although an effect due to alterations on both, AMPA and kainate receptors cannot be excluded by the results). In contrast, in the aRSC only the CNQX induced larger latencies in *Lis1/sLis1* slices, which suggest that in this cortical area the increased in latency observed in *Lis1/sLis1* animals could be caused mostly by alterations in the kainate receptor mediated transmission. The larger average peak amplitude of the sEPSC's recorded in the aRSC should be against a decreased velocity of propagation of EA, but the increased frequency indicates a facilitated synaptic transmission, which should increase the propagation velocity. The final propagation velocity should be the result of the balance of this two changes, without ruling out other mechanisms, such as alterations in the summing of synaptic responses in neurons). We do not have a clear explanation of the lack of effect of CTZ on the latencies of the propagating EA, but this finding could indicate that the strong inhibition of the desensitization of AMPA receptors caused by CTZ (Fucile et al. 2006) do not play a role in the propagation of electrical activity in this brain region, although CTZ reduce the latency of propagated activity in the spinal cord (Bonnot et al. 2009). The levels of glutamate receptors expressing the GluA2 subunit were not altered in the *Lis1/sLis1* cortex, as shown by immunohistochemistry and western blot; this finding discards a change in the level of these receptors as the cause of the differences in propagation of EA, but other possibilities remain open.

Finally, other interesting point is the heterogeneous phenotypical severity along the cingulate cortex. Data from latency of propagation (longer latencies in the contralateral hemispheres only in RSC) and possible glutamate receptor types implicated reflect that the posterior cingulate region (the retrosplenial cortex) displays a different phenotype in the *Lis1/sLis1* mouse. An explanation for this fact could be that LIS1 function might be more relevant in caudal regions, given that during cortical development, LIS1 is more expressed in caudal than in rostral regions (Escámez et al. 2012). These two areas of the cingulate cortex present some differences in structure and connectivity (Vogt and Paxinos 2012) and, therefore, they could be differentially affected.

Our main findings in the disinhibited cingulate cortex of *Lis1/sLis1* mice show a normal overall excitability and the presence of alterations in the epileptiform activity (a decreased frequency of oscillatory post-discharges and a slow propagation along layer 2/3); these findings suggest the presence of some type of network alteration since the intrinsic electrophysiological properties of the pyramidal neurons were not altered. Our data about glutamate receptors point to alterations of AMPA/kainate receptors that should be studied further, but alterations of other circuit mechanisms should also be investigated.

The above results and conclusions were obtained in a model lacking the LisH domain similar to a human case of lissencephaly (Fogli et al. 1999). Although early clinical studies suggest a certain genotype–phenotype relationship among LIS1 alterations and clinical profiles (Cardoso et al. 2000), this has been discarded by more accurate studies (Saillour et al. 2009; Uyanik et al. 2007). In fact, in the largest clinical characterization of lissencephalic patients (Saillour et al. 2009), authors described diverse LisH alterations in patients presenting a wide range of phenotypical profiles. It has been suggested by some authors that partial deletions and punctual mutations in the LisH domain might be related to less severe forms of lissencephaly (Cardozo et al. 2000), although that observation is debated (Saillour et al. 2009). According to that, our results revealed that, although the LisH domain deletion have some relevant functional consequences in the neuronal networks (properties and propagation of EA), other functional properties were not affected in contrast to what has been described in the Lis ± mouse model. For instance, enhanced excitability and epileptic activity observed in the Lis ± model (Greenwood et al. 2009), are absent in the *Lis1/sLis1* model. Interestingly, the mutation of the *Lis1/sLis1* model mimics the mutation presented by a patient with a mild lissencephalic phenotype (Fogli et al. 1999). Thus, our results point to the relationship among LisH domain alterations and less severe forms of lissencephaly. Furthermore, the fact that a homologous mutation produces a less severe phenotype in humans and also in mice suggests that this genotype–phenotype relationship might be preserved across the mammalian phylogeny, supporting the utility of murine models for this purpose.

We conclude that in the *Lis1/sLis1* model the cingulate cortex has a normal overall excitability, but the epileptiform discharges evoked in conditions of disinhibition have altered properties (a lower frequency of oscillatory post-discharges) and propagate along layer 2/3 at a lower velocity. Our findings about the slower propagation of EA and the possible implication of glutamate receptors could shed light on the implication of the gene LIS1 on complex electrical responses that are generated by cortical circuits; however, given that human lissencephaly is strongly determined by cortical structural abnormalities (and our model lacks those abnormalities) it is difficult to relate our findings to those pathophysiological mechanisms underlying the clinical symptoms of lissencephaly.

## Data Availability

The datasets generated during and/or analyzed during the current study are not publicly available, but are available from the corresponding author on reasonable request.
